# Quantum coherence of a circularly accelerated atom in a spacetime with a reflecting boundary

**DOI:** 10.1038/s41598-022-16647-9

**Published:** 2022-07-22

**Authors:** Wanhe Zhang, Xiaobao Liu, Tingli Yang

**Affiliations:** 1 Department of Innovation and Entrepreneurship, Liupanshui Normal University, Liupanshui, 553004 Guizhou China; 2 Department of Physics and Electrical Engineering, Liupanshui Normal University, Liupanshui, 553004 Guizhou China

**Keywords:** Quantum information, Quantum mechanics, Theoretical physics

## Abstract

We investigate, in the paradigm of open quantum systems, the dynamics of quantum coherence of a circularly accelerated atom coupled to a bath of vacuum fluctuating massless scalar field in a spacetime with a reflecting boundary. The master equation that governs the system evolution is derived. *Our results show that in the case without a boundary, the vacuum fluctuations and centripetal acceleration will always cause the quantum coherence to decrease. However, with the presence of a boundary, the quantum fluctuations of the scalar field are modified, which makes that quantum coherence could be enhanced as compared to that in the case without a boundary.* Particularly, when the atom is very close to the boundary, although the atom still interacts with the environment, *it behaves as if it were a closed system and* quantum coherence can be shielded from the effect of the vacuum fluctuating scalar field.

## Introduction

Quantum coherence, introduced by the superposition principle of quantum states^1^, plays the key role in quantum theory and quantum technology such as quantum optics^2,3^, quantum information^4^, solid-state physics^5,6^ as well as biology systems^7–12^, and so on. In this respect, several important works was proposed in order to develop a rigorous theory of coherence as a physical resource^13,14^ and put forward the necessary constraints to assess valid quantifiers of coherence^15^. Hence, in a recent work, Baumgratz et al.^16^ proposed a rigorous framework to quantify quantum coherence such as $l_1$ norm of coherence and relative entropy of coherence. *The point should be emphasized is that these two coherence measures have different physical interpretations. As shown in Refs.*^17–20^, *the* $l_1$ *norm of coherence acts as a good quantifier which captures the wave nature of a quanton in a multipath quantum interference scenario and it could be possible to experimentally detectable. Moreover, the relative entropy of coherence denotes the optimal rate of the distilled maximally coherent states that can be transformed by incoherent operations as the number of copies goes to infinity*^20,21^. *Recently, a lot of attentions have been focused on the research of resource theory of coherence, and this resource has been applied to various fields*^22,23^.

On the other hand, since every realistic system will unavoidably suffer from the decoherence and noise induced by the external environment, many ways were developed to enhance or protect the quantum resources, as the authors do when they analyze quantum correlation and metrology^24–30^. *Moreover, there have been sufficiently investigated in Refs.*^31–36^ *that suitable non-Markovian structured environments can efficiently preserve quantum coherence and entanglement.* Therefore, in Refs.^37,38^ we discussed quantum coherence of a *inertial* atom coupled to the fluctuating electromagnetic field, and it can be protected with the presence of boundaries. Another example is related to investigations of quantum coherence for the *accelerated* atom immersed in electromagnetic field with a boundary^39^. This was also the subject of study by the author in Ref.^40–42^. Inspired by these works, we find that quantum coherence of a two-level atom moving with a more realistic trajectory is worth discussed, i.e., the atom moves in a uniform circular motion, since the very large acceleration which is required for experiments is easier to achieve in circular motion.

In the present paper, we plan to study the quantum coherence *, measured by the* $l_1$ *norm of coherence and the relative entropy of coherence,* of a circularly accelerated atom coupled with the massless scalar field in analogy with the electric dipole interaction, as considered in Ref.^43^. It is worth mentioning that quantum coherence as a quantum resource decreases with the evolution time, which is due to the interaction between the atom and scalar field. Therefore, in order to enhance or even protect the quantum coherence, we would like to investigate the modification of the dynamics of quantum coherence by the presence of a boundary. In contrast to the case of without a boundary, our results show that as the atom gets closer and closer to the boundary, quantum coherence can be enhanced and may even be shielded from the influence of the external environment as if it were a closed system. The organization of the paper is as follows. In “Preliminaries”, we introduce the way to quantify quantum coherence and derive the master equation that the system obeys. In “Quantum coherence of a circularly accelerated atom near a conducting plate”, we calculate in detail quantum coherence of a circularly accelerated atom interacting with the massless scalar field in the presence of a reflecting boundary and we also make a comparison between our results and those of the unbounded case. A summary is given in Sec. IV. In this paper we use units $\hbar =c=k_B=1$.

## Preliminaries

In this approach taken in Ref.^16^, quantum coherence can be measured in the reference basis which is due to the off-diagonal elements of a density matrix $\rho$, for instance, the $l_1$ norm of coherence *and the relative entropy of coherence. Mathematically, these two coherence measures are defined as*

1 $$\begin{aligned} C_{l_1}(\rho )=\sum _{\begin{array}{c} i,j\\ i\ne j \end{array}}|\rho _{i,j}|, \end{aligned}$$

and

2 $$\begin{aligned} C_{\rm{RE}}(\rho )=S(\rho _{\rm{diag}})-S(\rho ), \end{aligned}$$

*respectively. Here,* $S(\rho )=-{\textbf {Tr}}(\rho \log \rho )$ *is the von Neumann entropy, and* $\rho _{\rm{diag}}$ *is the matrix only containing the diagonal elements of* $\rho$.

In quantum sense, any system should be regarded as an open system due to the interaction between the system and its surrounding environments. We consider the model which is consisted of a circularly accelerated atom interacting with a bath of fluctuating massless scalar field in the Minkowski vacuum. The total Hamiltonian of the atom-field system is

3 $$\begin{aligned} H=H_A+H_{\Psi }+H_I. \end{aligned}$$

Here, $H_A=\frac{1}{2}\omega _0\sigma _z$ denotes the Hamiltonian of atom, with $\omega _0$ being the energy-level spacing of the atom and $\sigma _z$ being the Pauli matrix, and $H_{\Psi }$ is the Hamiltonian of scalar field. We assume the the coupling between the detector and the massless scalar field is weak and their interaction Hamiltonian $H_I$, which is in analogy to the electric dipole interaction^44^,

4 $$\begin{aligned} H_I=\mu (\sigma _+ + \sigma _-)\Psi (x(\tau )), \end{aligned}$$

with $\mu$ being the coupling constant that we assume to be small, $\sigma _+$ ($\sigma _-$) being the rasing (lowering) operator of the detector, and $\Psi (x(\tau ))$ corresponding to the scalar field operator with $\tau$ being the detector’s proper time.

At the beginning, the total density operator of the atom-field system can be represented as $\rho _{tot}=\rho _A(0)\otimes |0\rangle \langle 0|$, in which $\rho _A(0)$ is the initial reduced density matrix of the atom and $|0\rangle$ represents the vacuum for the massless scalar field. The equation of motion of the whole system in the interaction picture can be described by,

5 $$\begin{aligned} \frac{\partial \rho _{tot}(\tau )}{\partial \tau }= & {} -i[H_I(\tau ),\rho _{tot}(\tau )]. \end{aligned}$$

With the help of $\rho _{tot}(\tau )=\rho _{tot}(0)-i\int ^\tau _0ds[H_I(s),\rho _{tot}(s)]$, by taking the partial trace over the environmental degrees of freedom and $Tr_B[H_I(\tau ),\rho _{tot}(0)]=0$, the Eq. (5) can be rewritten as

6 $$\begin{aligned} \frac{\partial \rho _A(\tau )}{\partial \tau }=-\int _0^\tau dsTr_B[H_I(\tau ),[H_I(s),\rho _{tot}(s)]]. \end{aligned}$$

Now, we assume that atom and field are weakly coupled (i.e., Born approximation^45^). This approximation is equivalent to assuming that the correlations established between atom and field are negligible at all times (initially zero), namely:

7 $$\begin{aligned} \rho _{tot}(s)\approx \rho _A(s)\otimes \rho _B. \end{aligned}$$

Furthermore, we introduce the second approximation, the Markov approximation^45^, which states that the bath has a very short correlation time $\tau _B$. If $\tau \gg \tau _B$, we can replace $\rho _A(s)$ by $\rho _A(\tau )$, since the short “memory” of the bath correlation function causes it to keep track of events only within the short period $[0,\tau _B]$. Moreover, for the same reason we can extend the upper limit of the integral in Eq. (6) to infinity without changing the value of the integral. Therefore, with the help of Eq. (7), we have

8 $$\begin{aligned} \frac{\partial \rho _A(\tau )}{\partial \tau }=-\int _0^\infty ds\,Tr_B[H_I(\tau ),[H_I(s),\rho _A(\tau )\otimes \rho _B]].\nonumber \\ \end{aligned}$$

Inserting Eq. (4) into Eq. (8), we can get the master equation in the Kossakowski-Lindblad form^46–48^

9 $$\begin{aligned} \frac{\partial \rho _A(\tau )}{\partial \tau }= & {} -i[H_{eff},\rho _A(\tau )]\nonumber \\&+\sum ^3_{j=1}[2L_j\rho _A L^\dagger _j-L^\dagger _jL_j\rho _A-\rho _A L^\dagger _jL_j], \end{aligned}$$

where $H_{eff}$ and $L_j$ are given by

10 $$\begin{aligned}{}&H_{eff}=\frac{1}{2}\Omega \sigma _z=\frac{1}{2}\{\omega _0+\mu ^2\mathrm {Im}(\Gamma _++\Gamma _-)\}\sigma _z\;,\nonumber \\&L_1=\sqrt{\frac{\gamma _-}{2}}\sigma _-\;,L_2=\sqrt{\frac{\gamma _+}{2}}\sigma _+\;,L_3=\sqrt{\frac{\gamma _z}{2}}\sigma _z, \end{aligned}$$

with

11 $$\begin{aligned} \gamma _{\pm }= & {} 2\mu ^2\mathrm{Re}\Gamma _\pm =\mu ^2\int ^{+\infty }_{-\infty }e^{\mp i\omega _0\triangle \tau }G^+(s- i\epsilon )d\triangle \tau ,\nonumber \\ \gamma _{z}= & {} 0, \end{aligned}$$

in which $s=\tau -\tau '$, $G^+(x-x')=\langle 0|\Psi (x)\Psi (x')|0\rangle$ is the two-point correlation function of the massless scalar field with $x\equiv x(\tau )$ and $x'\equiv x(\tau ')$^49^.

Assume that the initial state of two-level atom is a maximal coherent state $|\phi (0)\rangle = \frac{1}{\sqrt{2}}(|0\rangle + |1\rangle )$. Then, according to Eq. (9), the corresponding time-dependent reduced density matrix can be obtained as

12 $$\begin{aligned} \rho (\tau )=\frac{1}{2} \left( \begin{array}{cc} 1+ \frac{\gamma _+ - \gamma _-}{\gamma _+ + \gamma _-}[1-e^{-(\gamma _+ + \gamma _-)\tau }]&{} e^{-\frac{1}{2}(\gamma _+ + \gamma _-)\tau -i\Omega \tau }\\ e^{-\frac{1}{2}(\gamma _+ + \gamma _-)\tau +i\Omega \tau }&{}1-\frac{\gamma _+ - \gamma _-}{\gamma _+ + \gamma _-}[1-e^{-(\gamma _+ + \gamma _-)\tau }] \end{array} \right) . \end{aligned}$$

In the above equation, we note that $\frac{1}{2}(\gamma _+ + \gamma _-)$ is the time scale for the off-diagonal elements of the density-matrix (“coherence”) decay and $\gamma _+ + \gamma _-$ represents the time scale for atomic transition^50^.

## Quantum coherence of a circularly accelerated atom near a conducting plate

We now investigate the quantum coherence of an atom rotating in the $x-y$ plate a distance $z_0$ from the boundary. The plate is located at $z=0$. Our approach generalizes the method developed by Takagi^51^ to the case when boundary conditions are present. In the Minkowski coordinate, the world line of the circular motion of radius *R* at a constant speed $\upsilon$ with centripetal acceleration $a=\frac{\gamma ^2\upsilon ^2}{R}$ is given by

13 $$\begin{aligned} t(\tau )= & {} \gamma \tau ,\nonumber \\ x(\tau )= & {} R\cos \omega \gamma \tau , \nonumber \\ y(\tau )= & {} R\sin \omega \gamma \tau ,\nonumber \\ z(\tau )= & {} z_0\;, \end{aligned}$$

where $\omega$ is the angular velocity

14 $$\begin{aligned} \omega =\upsilon /R, \end{aligned}$$

and $\gamma$ is the Lorentz factor

15 $$\begin{aligned} \gamma =(1-\upsilon ^2)^{-1/2}. \end{aligned}$$

The parameter $\tau$ is the proper time as usual.

In order to obtain the quantum coherence of atom in the presence of a boundary, we first calculate the correlation function of the scalar field $G^{+}(x-x')$ consisted of a sum of two terms, i.e., an empty-space contribution $G^{+}(x-x')_0$ and a term $G^{+}(x-x')_R$ which is the correction induced by the presence of the plate with Dirichlet boundary conditions^49,52^

16 $$\begin{aligned} G^{+}(x-x')=G^{+}(x-x')_0+G^{+}(x-x')_R, \end{aligned}$$

where

17 $$\begin{aligned}{}&G^{+}(x-x')_0=\frac{1}{4\pi ^2}\nonumber \\&\;\;\;\;\times \frac{1}{(x-x')^2+(y-y')^2+(z-z')^2-(t-t'-i\epsilon )^2}, \end{aligned}$$

and

18 $$\begin{aligned}{}&G^{+}(x-x')_R=-\frac{1}{4\pi ^2}\nonumber \\&\;\;\;\;\times \frac{1}{(x-x')^2+(y-y')^2+(z+z')^2-(t-t'-i\epsilon )^2}. \end{aligned}$$

According to the trajectories of the atom (13), one can easily get the correlation function as

19 $$\begin{aligned} G^{+}(x-x')= & {} -\frac{1}{4\pi ^2}\frac{1}{\gamma ^2(\triangle \tau -i\epsilon )^2-(\frac{2\upsilon ^2\gamma ^2}{a})^2\sin ^2(\frac{a\triangle \tau }{2\upsilon \gamma })}\nonumber \\&+\frac{1}{4\pi ^2}\frac{1}{\gamma ^2(\triangle \tau -i\epsilon )^2-(\frac{2\upsilon ^2\gamma ^2}{a})^2\sin ^2(\frac{a\triangle \tau }{2\upsilon \gamma })-4z_0^2}, \end{aligned}$$

which can be alternatively written as

20 $$\begin{aligned} G^{+}(x-x')= & {} -\frac{1}{4\pi ^2}\frac{1}{ (\triangle \tau -i\epsilon )^2[1+f(\triangle \tau )]}\nonumber \\&+\frac{1}{4\pi ^2}\frac{1}{(\triangle \tau -i\epsilon )^2[1+f(\triangle \tau )]-4z_0^2}, \end{aligned}$$

with

21 $$\begin{aligned} f(\triangle \tau )=\frac{a^2\triangle \tau ^2}{12}-\frac{a^4\triangle \tau ^4}{360\upsilon ^2\gamma ^2}+.... \end{aligned}$$

Here, we expand $\sin ^2(\frac{a\triangle \tau }{2\upsilon \gamma })= \frac{a^2\triangle \tau ^2}{4\upsilon ^2\gamma ^2}-\frac{a^4\triangle \tau ^4}{48\upsilon ^4\gamma ^4}+\frac{a^6\triangle \tau ^6}{1440\upsilon ^6\gamma ^6}+...$ with $\triangle \tau =\tau -\tau '$. As is hard to find the explicit form of $\gamma _+$ and $\gamma _-$, we now consider the ultrarelativistic limit, i.e., $\gamma \gg 1$, shown in Ref.^53^, so the field correlation function becomes

22 $$\begin{aligned} G^{+}(x-x')= & {} -\frac{1}{4\pi ^2}\frac{1}{(\triangle \tau -i\epsilon )^2[1+\frac{a^2\triangle \tau ^2}{12}]}\nonumber \\&+\frac{1}{4\pi ^2}\frac{1}{(\triangle \tau -i\epsilon )^2[1+\frac{a^2\triangle \tau ^2}{12}]-4z_0^2}. \end{aligned}$$

Then, the Fourier transform of the field correlation function, which corresponds to the spontaneous emission rate, is

23 $$\begin{aligned} \gamma _-= & {} \gamma _0\bigg [1+\frac{a}{4\sqrt{3}}e^{-2\sqrt{3}\frac{\omega _0}{a}}\nonumber \\&-\frac{\sqrt{3}ae^{-\frac{\omega _0}{a}\sqrt{6+2\sqrt{9+12a^2z_0^2}}}}{2\sqrt{(3+\sqrt{9+12a^2z_0^2})(6+8a^2z_0^2)}\omega _0}\nonumber \\&-\frac{\sqrt{3}a\sin \bigg (\frac{\omega _0}{a}\sqrt{-6+2\sqrt{9+12a^2z_0^2}}\bigg )}{\sqrt{(-3+\sqrt{9+12a^2z_0^2})(6+8a^2z_0^2)}\omega _0}\bigg ], \end{aligned}$$

where $\gamma _0=\frac{\omega _0\mu ^2}{2\pi }$ denotes the spontaneous emission rate for the atom coupled with scalar field without boundary. Similarly, the spontaneous excitation rate is given by

24 $$\begin{aligned} \gamma _+= & {} \gamma _0\bigg [\frac{a}{4\sqrt{3}}e^{-2\sqrt{3}\frac{\omega _0}{a}}\nonumber \\&-\frac{\sqrt{3}ae^{-\frac{\omega _0}{a}\sqrt{6+2\sqrt{9+12a^2z_0^2}}}}{2\sqrt{(3+\sqrt{9+12a^2z_0^2})(6+8a^2z_0^2)}\omega _0}\bigg ], \end{aligned}$$

Inserting Eqs. (23) and (24) into Eq. (12), the $l_1$ *norm of coherence* (1) *and the relative entropy of coherence* (2) for the atom in the presence of a boundary are found to be

25 $$\begin{aligned} C_{l_{1}}(\tau )= e^{-\frac{1}{2}f(\omega _0,a,z_0)\gamma _0\tau }, \end{aligned}$$

and

26 $$\begin{aligned} C_{\rm{RE}}(\tau )= & {} -M\log _{2}M-(1-M)\log _{2}(1-M) \nonumber \\&+\lambda _{+}\log _{2}\lambda _{+} +\lambda _{-}\log _{2}\lambda _{-}, \end{aligned}$$

where

27 $$\begin{aligned} M=\frac{1}{2}\bigg [1+ \frac{g(\omega _0,a,z_0)}{f(\omega _0,a,z_0)}[1-e^{-f(\omega _0,a,z_0)\gamma _0\tau }]\bigg ], \end{aligned}$$

28 $$\begin{aligned} \lambda _{\pm }=\frac{1}{2}\pm \sqrt{\frac{1}{4}e^{-f(\omega _0,a,z_0)\gamma _0\tau }+(M-\frac{1}{2})^2}. \end{aligned}$$

*In the above equations, for simplify, we let*

29 $$\begin{aligned} g(\omega _0,a,z_0)= & {} -1+\frac{\sqrt{3}a\sin \bigg (\frac{\omega _0}{a}\sqrt{-6+2\sqrt{9+12a^2z_0^2}}\bigg )}{\sqrt{(-3+\sqrt{9+12a^2z_0^2})(6+8a^2z_0^2)}\omega _0}, \end{aligned}$$

and

30 $$\begin{aligned} f(\omega _0,a,z_0)= & {} 1+\frac{a}{2\sqrt{3}\omega _0}e^{-\frac{2\sqrt{3}\omega _0}{a}}\nonumber \\&-\frac{\sqrt{3}ae^{-\frac{\omega _0}{a}\sqrt{6+2\sqrt{9+12a^2z_0^2}}}}{\sqrt{(3+\sqrt{9+12a^2z_0^2})(6+8a^2z_0^2)}\omega _0} \nonumber \\&-\frac{\sqrt{3}a\sin \bigg (\frac{\omega _0}{a}\sqrt{-6+2\sqrt{9+12a^2z_0^2}}\bigg )}{\sqrt{(-3+\sqrt{9+12a^2z_0^2})(6+8a^2z_0^2)}\omega _0}. \end{aligned}$$

Comparing the above results with Eq. (25) of Ref.^28^, we can see that the function $f(\omega _0,a,z_0)$ gives the modification induced by the presence of the boundary. Here, $\gamma _R=f(\omega _0,a,z_0)\gamma _0$ represents the spontaneous emission rate for the circularly accelerated atom with a boundary. Note that for the centripetal acceleration $a/\omega _0\rightarrow 0$, we have $f(\omega _0,a,z_0)=1-\frac{\sin 2\omega _0z_0}{2\omega _0z_0}$ and find that the transition rate recovers to that of an inertial atom interacting with the massless scalar field with a boundary^54^.

Before the investigate of the whole evolution process, let us first examine that when evolving long enough time, i.e., $\tau \gg \frac{1}{\gamma _+ + \gamma _-}$ with $\frac{1}{\gamma _+ + \gamma _-}$ being the time scale for atomic transition, the system thermalizes to the steady state

31 $$\begin{aligned} \rho (\infty )=\frac{1}{\gamma _+ + \gamma _-} \left( \begin{array}{cc} \gamma _+ &{} 0\\ 0&{}\gamma _- \end{array} \right) . \end{aligned}$$

We remark that the steady state in Eq. (31) is independent of the initial state, and the quantum coherence vanishes, namely: $C_{l_{1}}(\infty )=0$ and $C_\mathrm{RE}(\infty )=0$. This indicates that quantum coherence does not maintain for a long time under the effect of vacuum fluctuating scalar field.

**Figure 1:**
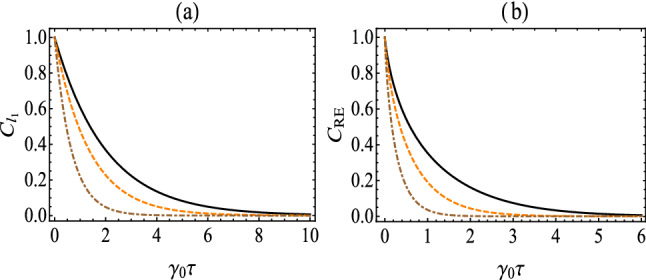
(Color online) The $l_1$ norm of coherence (**a**) and the relative entropy of coherence (**b**) as a function of $\gamma _0\tau$ for the case without boundary. The solid, dashed and dotted-dashed lines correspond to $a/\omega _0=0.1$, $a/\omega _0=4$ and $a/\omega _0=10$, respectively.

Now let us examine the asymptotic behaviors of quantum coherence, i.e., when the atom is placed very close to the boundary $(\omega _0z_0\rightarrow 0)$ or very far from it $(\omega _0z_0\rightarrow \infty )$. When $\omega _0z_0\rightarrow 0$, $f(\omega _0,a,z_0)=0$ and $g(\omega _0,a,z_0)=0$, one has $C_{l_{1}}(\tau )=1$ and $C_{\rm{RE}}(\tau )=1$. This means that as the atom very closes to the boundary, quantum coherence is shield from the influence of the scalar field as if it were isolated. While for the case when $\omega _0z_0\rightarrow \infty$, $f(\omega _0,a,z_0)\rightarrow 1+\frac{a}{2\sqrt{3}\omega _0}e^{-\frac{2\sqrt{3}\omega _0}{a}}$ and $g(\omega _0,a,z_0)\rightarrow -1$, our results reduce to those of the unbounded Minkowski space^28,43^ as expected. For the unbound case, as shown in Fig. 1, quantum coherence, i.e., the $l_1$ *norm of coherence and the relative entropy of coherence,* decreases with the evolution time, due to the fact that the decoherence is caused by the interaction between the atom and massless scalar field. Additionally, we find that as the centripetal acceleration $a/\omega _0$ increases, which makes quantum coherence decay faster.

**Figure 2:**
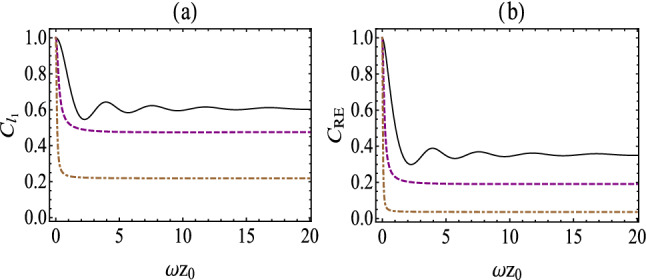
(Color online) $C_{l_{1}}(\tau )$ **(a) ** and $C_\mathrm{RE}(\tau )$ **(b)** as a function of $\omega _0 z_0$ for the fixed value $\gamma _0\tau =1$ in the presence of a boundary. The solid, dashed and dotted-dashed lines correspond to $a/\omega _0=0.1$, $a/\omega _0=4$, $a/\omega _0=10$, respectively.

**Figure 3:**
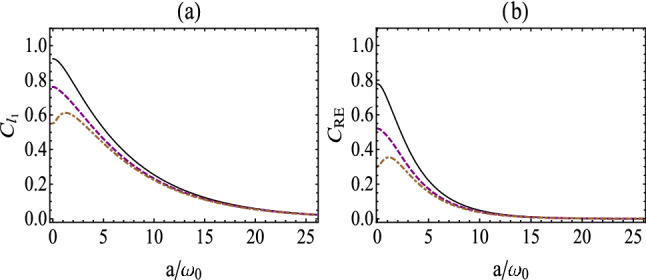
(Color online) $C_{l_{1}}(\tau )$ **(a)** and $C_\mathrm{RE}(\tau )$ **(b)** as a function of $a/\omega _0$ for the fixed value $\gamma _0\tau =1$ in the presence of a boundary. The solid, dashed and dotted-dashed lines correspond to $\omega _0 z_0=0.5$, $\omega _0 z_0=1$, $\omega _0 z_0=2$, respectively.

For a generic case, the dynamics of quantum coherence are dependent on the evolution time, boundary effects and the centripetal acceleration. As shown in Figs. 2 and 3, we plot quantum coherence, i.e., the $l_1$ *norm of coherence and the relative entropy of coherence,* as a function of the atomic position $\omega _0 z_0$ (centripetal acceleration $a/\omega _0$) with different centripetal acceleration (atomic position). Here, we take the fixed value $\gamma _0\tau =1$. From Fig. 2, we find that quantum coherence saturates at different minimum values for different centripetal acceleration in the limit of infinite atomic position. However, we can see that for small centripetal acceleration, the quantum coherence fades to a stable value in an oscillatory manner. Also, in Fig. 2 we note that the maximal value of quantum coherence is obtained when $\omega _0z_0\rightarrow 0$, i.e., $C_{l_{1}}(\tau )=1$ and $C_{l_{1}}(\mathrm RE)=1$, which implies that quantum coherence is immune to the external environment[refer to the case for the atom placed very close to the boundary]. Besides, Fig. 3 presents that quantum coherence decreases and reduces to zero in the limit of infinite centripetal acceleration. While for large atomic position, quantum coherence will increases for a while and starts to decrease to zero. This implies that quantum coherence can be enhanced by centripetal acceleration under some circumstances. *Furthermore, we can see from Figs.* 2 and 3*that quantum coherence measured by the relative entropy of coherence fall faster than the same measured by the* $l_1$ *norm of coherence, which is similar to the results of Ref.*^20^.

**Figure 4:**
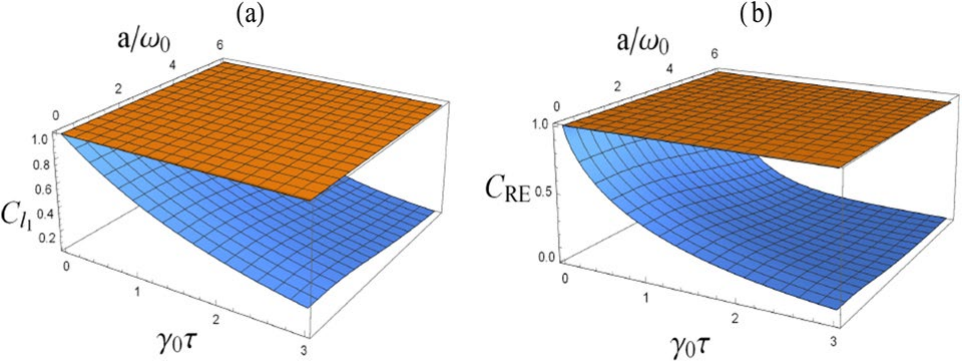
(Color online) Comparison between quantum coherence, i.e., $C_{l_{1}}(\tau )$ (**a**) and $C_{\rm{RE}}(\tau )$ (**b**), for the case of with the presence of boundary (the top yellow surface) and the case of without a boundary (the bottom blue surface), with $\omega _0 z_0\rightarrow 0$.

**Figure 5:**
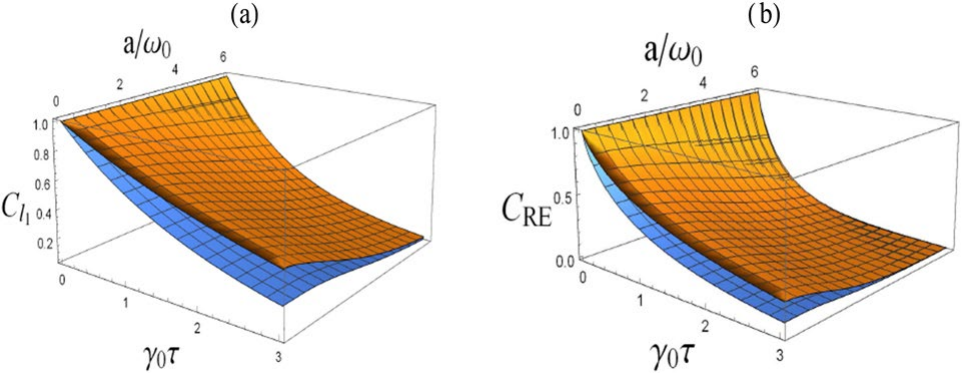
(Color online) Comparison between quantum coherence, i.e., $C_{l_{1}}(\tau )$ (**a**) and $C_{\rm{RE}}(\tau )$ (**b**), for the case of with the presence of boundary (the top yellow surface) and the case of without a boundary (the bottom blue surface), with $\omega _0 z_0= 1$.

More importantly, to compare quantum coherence of the atom with and without the presence of a boundary, we plot, in Figs. 4 and 5, quantum coherence with respect to evolution time and centripetal acceleration, for different values of atomic position, i.e., $\omega _0 z_0\rightarrow 0$ and $\omega _0 z_0=1$ respectively. It is obvious that for the case of without a boundary, quantum coherence decreases by increasing the value of evolution time and centripetal acceleration. However, with the presence of a boundary, as we can see from Fig. 4, when the atom very close to the boundary, i.e., $\omega _0z_0\rightarrow 0$, quantum coherence always closes to 1. That is, quantum coherence *, measured by the* $l_1$ *norm of coherence and the relative entropy of coherence,* is shielded from the influence of the vacuum fluctuations of the massless scalar field when the atom is close to the boundary. Besides, in Fig. 5, when $\omega _0 z_0=1$, despite of quantum coherence decreasing as the time and centripetal acceleration grow, while in contrast to the unbounded case, quantum coherence decays slowly in the case of a boundary. This means that quantum coherence*, measured by the* $l_1$ *norm of coherence and the relative entropy of coherence,* can be enhanced in some degree with a boundary. As a result, we argue that as the atom gets closer and closer to the boundary, quantum coherence, i.e., the $l_1$ *norm of coherence and the relative entropy of coherence,* can be enhanced or even shielded from the influence of environment by the presence of boundary.

## Conclusion

In this letter, we have studied the dynamics of quantum coherence*, measured by the* $l_1$ *norm of coherence and the relative entropy of coherence,* of a circularly accelerated two-level atom in a space with a reflecting boundary in the framework of open quantum systems. Assuming a dipole-like interaction between the atom and a scalar field, the master equation that describe the system evolution is derived. In the case without a boundary, it is found that quantum coherence decreases with respect to the time, due to the fact that the interaction between the atom and scalar field. Also, a decreasing quantum coherence is observed as centripetal acceleration increases. In the case with a boundary, when the atomic distance far from the boundary $\omega _0 z_0\rightarrow \infty$, the corrections induced by the presence of a boundary become negligible as one would expect, which means that the behaviors of quantum coherence recover to the results obtained for the *case without* a boundary. However, when the atom close to the boundary, we found that quantum coherence decreases slowly, which implies that quantum coherence will be enhanced as compared to the case without any boundary. More remarkably, we are interested to note that when the atom very close to the boundary $\omega _0 z_0\rightarrow 0$, the modifications induced by the presence of a boundary become so large that quantum coherence can be shielded from the influence of the vacuum fluctuating scalar field.

## Data Availability

The data that support the plots within this paper and other findings of this study are available from the corresponding author upon request.
